# The spatial separation of basic amino acids is similar in RHAMM and hyaluronan binding peptide P15‐1 despite different sequences and conformations

**DOI:** 10.1002/pgr2.70001

**Published:** 2024-09-10

**Authors:** Mehmet Emre Erkanli, Ted Keunsil Kang, Thorsten Kirsch, Eva A. Turley, Jin Ryoun Kim, Mary K. Cowman

**Affiliations:** ^1^ Department of Chemical and Biomolecular Engineering, Tandon School of Engineering New York University Brooklyn New York USA; ^2^ Department of Biomedical Engineering, Tandon School of Engineering New York University New York New York USA; ^3^ Department of Orthopedic Surgery, Grossman School of Medicine New York University New York New York USA; ^4^ Verspeeten Family Cancer Centre, London Health Sciences Centre, Lawson Health Research Institute London Ontario Canada; ^5^ Departments of Oncology, Biochemistry and Surgery, Schulich School of Medicine and Dentistry Western University London Ontario Canada

**Keywords:** hyaluronan, P15‐1, peptide conformation, RHAMM, therapeutic peptide

## Abstract

Peptides that increase pro‐reparative responses to injury and disease by modulating the functional organization of hyaluronan (HA) with its cell surface binding proteins (e.g., soluble receptor for hyaluronan‐mediated motility [RHAMM] and integral membrane CD44) have potential therapeutic value. The binding of RHAMM to HA is an attractive target, since RHAMM is normally absent or expressed at low levels in homeostatic conditions, but its expression is significantly elevated in the extracellular matrix during tissue stress, response‐to‐injury, and in cancers and inflammation‐based diseases. The HA‐binding site in RHAMM contains two closely spaced sequences of clustered basic amino acids, in an alpha‐helical conformation. In the present communication, we test whether an alpha‐helical conformation is required for effective peptide binding to HA, and competitive disruption of HA–RHAMM interaction. The HA‐binding RHAMM‐competitive peptide P15‐1, identified using the unbiased approach of phage display, was examined using circular dichroism spectroscopy and the conformation‐predictive AI‐based AlphaFold2 algorithm. Unlike the HA‐binding site in RHAMM, peptide P15‐1 was found to adopt irregular conformations in solution rather than alpha helices. Instead, our structural analysis suggests that the primary determinant of peptide‐HA binding is associated with a specific clustering and spacing pattern of basic amino acids, allowing favorable electrostatic interaction with carboxylate groups on HA.

**Abbreviations**
aa: amino acid
BX_7_B: a nine amino acid sequence containing terminal basic (B) amino acids separated by internal (X) amino acids that are not acidic, and include at least one basic amino acid
CD: circular dichroism
HA: hyaluronan
HA8: HA octasaccharide
P15‐1: peptide sequence STMMSRSHKTRSHHV
Pep‐1: peptide sequence GAHWQFNALTVR
pLDDT: predicted local distance difference test
RG peptide: peptide sequence RGGGRGRRR
RHAMM: receptor for hyaluronan‐mediated motility; CD168; gene name HMMR
ScrP15: peptide sequence HKSVSRHTSMRHSTM
YRHAMM1: peptide sequence YKQKIKHVVKLK

## INTRODUCTION

Extracellular interaction between the glycosaminoglycan hyaluronan (HA) and the protein RHAMM is a promising therapeutic target due to its instructive involvement in inflammation‐based disease, and the structural characteristics of effective peptide modulators are therefore of interest.

HA is a glycosaminoglycan with a repeating disaccharide structure of [(1→3)‐β‐d‐GlcNAc‐(1→4)‐β‐d‐GlcA]. In vertebrates, it is synthesized by integral membrane synthase enzymes HAS1, HAS2, and HAS3, which release growing chains directly into the extracellular space.^1^, ^2^ In healthy, homeostatic tissues, HA has a high molecular weight, averaging approximately 2000–6000 kDa.^3^

The primary cell surface receptor for HA is CD44, a constitutively expressed integral membrane glycoprotein^4^, ^5^ which is clustered under normal physiological conditions, in part by cooperative binding of the extracellular domains of multiple CD44 molecules to high molecular weight HA.^6^, ^7^, ^8^, ^9^ In contrast, when HA is fragmented by enzymes and/or reactive oxygen or nitrogen species,^10^ CD44 can become less extensively clustered.^8^ This results in increased susceptibility of CD44 to proteolytic cleavage, with shedding of the extracellular domain, and activation of pro‐inflammatory processes that are mediated by the liberated intracellular domain.^11^, ^12^, ^13^, ^14^, ^15^, ^16^, ^17^, ^18^, ^19^

HA also binds to the soluble protein RHAMM (receptor for hyaluronan‐mediated motility; CD168; gene name HMMR). Human RHAMM is a 724 amino acid protein.^20^ It is normally absent or expressed at low levels in homeostatic conditions, but its expression is transiently increased during tissue stress and response‐to‐injury.^21^ Under those conditions, RHAMM can be exported from the cytoplasm to the extracellular matrix by an unknown process,^22^, ^23^ where it binds to HA and co‐localizes with CD44.^21^, ^24^, ^25^, ^26^ Extracellular RHAMM stimulates migration of tumor cells,^27^ fibroblasts,^24^ and macrophages,^28^ and contributes to myofibroblast differentiation and fibrosis in remodeling tissue.^29^, ^30^ These effects of RHAMM require HA.^24^, ^31^ RHAMM also modulates other HA‐ and CD44‐dependent effects leading to aberrant cell signaling in inflammatory disease.^32^, ^33^, ^34^ Thus, a molecular agent capable of modulating HA binding to RHAMM in the presence of CD44 is of therapeutic interest.

**Figure 1:**
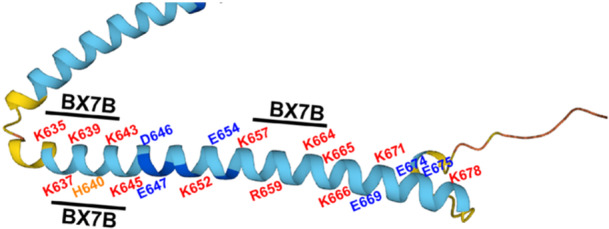
The hyaluronan (HA)‐binding region of human receptor for hyaluronan‐mediated motility (RHAMM) shows a high density of basic amino acid residues (labeled in red and orange), located in BX_7_B‐containing clusters, separated by a short segment containing several acidic residues (labeled in blue). The RHAMM segment depicted is a portion of the full‐length human RHAMM structure model predicted by AlphaFold, and downloaded from https://alphafold.ebi.ac.uk/entry/O75330 (Deep Mind and EMBL‐EBI).^37^, ^38^ The RHAMM chain is colored according to the confidence level of the predicted conformation (dark blue: very high; light blue: confident; yellow: low).

The site in RHAMM to which HA binds is localized to two closely spaced domains near the C‐terminus.^22^, ^35^, ^36^ The amino acid sequences of human RHAMM that are required for HA binding are K^635^QKIKHVVKLK^645^ and K^657^LRCQLAKKK^666^ (Figure 1). Both HA‐binding sequences contain BX_7_B motifs, where B is R or K in RHAMM, X is not acidic, and X should include at least one basic amino acid (Table 1). HA‐RHAMM binding is electrostatically stabilized and disrupted by 1 M NaCl,^35^, ^39^ and the BX_7_B motifs in the binding sequences suggest that the binding involves specific structural fit of the closely spaced basic amino acids with the carboxylates of HA. While the conformation of full‐length RHAMM has not yet been experimentally determined, the conformation of synthetic 60‐62 aa (ca. 7 kDa) fragments of the mouse RHAMM sequence containing both BX_7_B motifs are mainly alpha helical in water or dilute phosphate buffer, based on circular dichroism analysis.^40^, ^41^ Predominantly alpha‐helical conformations have been predicted for both full‐length human RHAMM (https://alphafold.ebi.ac.uk/entry/O75330) and mouse RHAMM (https://alphafold.ebi.ac.uk/entry/Q00547) by the AlphaFold AI system (Deep Mind and EMBL‐EBI).^37^, ^38^

**Table 1:** HA‐binding sequences of RHAMM and P15‐1 peptide.^a^

BX_7_B motif (HA binding)	*B* *XXXXXXX* *B* X not acidic, should have ≥1 basic
RHAMM (mouse)	*K* *Q* *K* *I* *KH* *VV* *K* LK‐DENSQLKSEVS‐ *K* *L* *R* *SQLV* *KR*K
	KQ *K* *I* *KH* *VV* *K* *L* *K* ‐DENSQLKSEVS‐ *K* *L* *R* *SQLV* *KR*K
RHAMM (human)	*K* *Q* *K* *I* *KH* *VV* *K* LK‐DENSQLKSEVS‐ *K* *L* *R* *CQLA* *KK*K
	KQ *K* *I* *KH* *VV* *K* *L* *K* ‐DENSQLKSEVS‐ *K* *L* *R* *CQLA* *KK*K
P15‐1	STMMS *R* *S* *HK* *T* *R* *S* *HH* V

^a^ For sequences in which two or more identified BX_7_B motifs exist and overlap, the same peptide sequence is shown again with alternative motif locations identified. The BX_7_B motif of each sequence is highlighted in italic.

A number of therapeutic candidate peptides that target HA‐RHAMM have been identified by rational design, based on the two HA‐binding BX_7_B sequences of RHAMM.^35^, ^36^, ^42^, ^43^, ^44^, ^45^, ^46^ In addition, a 15‐mer RHAMM‐mimetic peptide STMMSRSHKTRSHHV, P15‐1, was discovered using an unbiased phage display peptide library screened for peptides binding to HA but not chondroitin sulfate.^29^ This peptide has a BX_7_B motif, although three basic residues of the sequence are H rather than R or K as found in RHAMM (Table 1). There is substantial evidence for P15‐1 binding to HA, with a binding affinity (*K*_d_) of approximately 100 nM to 10 µM,^29^, ^47^ depending on pH, and for P15‐1 disruption of HA‐RHAMM interaction,^29^ while P15‐1's scrambled version (HKSVSRHTSMRHSTM) is less effective. P15‐1 does not disrupt HA binding to CD44,^29^ reflecting the different structures of the RHAMM versus CD44 HA binding sites. The P15‐1 peptide, but not the scrambled peptide, has been shown to have therapeutic properties in preclinical studies, such as non‐fibrotic wound healing in rat skin,^29^ targeting inflammation in the brain following traumatic injury,^48^, ^49^ reducing osteoarthritis progression in a mouse injury model,^47^ and non‐fibrotic regenerative repair of a full thickness injury to articular cartilage in a rabbit model.^50^

RHAMM binding to HA has been proposed to utilize the BX_7_B motif in an alpha‐helical conformation that places the side chains of the basic residues for maximal interaction with HA.^22^, ^35^, ^40^ In the present communication, we address the question of whether the P15‐1 peptide, or its scrambled version, adopts an alpha‐helical conformation in buffered saline solution, thus mimicking the conformation of the HA‐binding sequence in RHAMM protein, to effectively compete with RHAMM for binding to HA. Surprisingly, our results from circular dichroism (CD) spectroscopy and predictive models indicate that the peptide is conformationally disordered rather than alpha helical. Instead, the specific spacing of basic residues of P15‐1 in an irregular extended conformation is proposed to be responsible for its binding to HA and competition with RHAMM.

## MATERIALS AND METHODS

### Sample preparation

Peptide P15‐1 (STMMSRSHKTRSHHV) (purity 95.8%) and Scrambled P15‐1 (ScrP15; HKSVSRHTSMRHSTM) (purity 98.7%), each having a theoretical molecular weight of 1782 g/mol, were synthesized and analyzed by HPLC and mass spectrometry by Genscript Biotech Corporation. Dry peptide samples were dissolved in sterile phosphate‐buffered saline (PBS; Cytivia; SH30256.01) at a concentration of 1.6 mg/mL, and stored at 4°C before use.

### Circular dichroism (CD) spectroscopy

Circular dichroism (CD) spectra were recorded using a Jasco J‐810 spectropolarimeter, at 25°C. Spectroscopic parameters and sample concentrations were optimized to allow data acquisition over a wavelength range from 250 to 200 nm, using a spectral bandwidth of 1 nm, response time 1 s, and scan rate 10 nm/min, allowing six response periods per unit of spectral bandwidth. Spectral data were averaged from five scans. The quartz optical cell path length was 0.1 cm. Peptide sample concentration was optimally 0.2 mg/mL in PBS, but the spectral properties were essentially unchanged over a studied concentration range of 0.1–0.4 mg/mL. At 0.4 mg/mL, data were restricted to wavelengths greater than about 209 nm due to higher absorbance. The average residue weight per amino acid of 119 g/mol was used to calculate molar ellipticity. No smoothing was applied to the data. A baseline control spectrum of PBS was subtracted from each sample spectrum.

To correct for any difference in concentration of the two peptide solutions prepared based on the reported sample masses in tubes as supplied, UV spectra were recorded using Varian Cary 50 UV‐VIS spectrophotometer (Agilent Technologies) with the quartz cuvettes of 1 cm path length and the ratios of the absorbances at 210, 215, and 220 nm for the scrP15 and P15‐1 samples were averaged. The scrP15/P15‐1 concentration ratio was 1.22 ± 0.01, and this factor was used to scale the scrP15 CD spectrum to the same concentration as P15‐1.

Mixtures of peptides with hyaluronan (HA) were also analyzed. HA samples with low polydispersity and weight‐average molecular weights of 50 kDa (Select‐HA™ 50 K), 500 kDa (Select‐HA™ 500 K), and 1500 kDa were obtained from Hyalose LLC (for 50 and 500 kDa) and Lifecore Biomedical (for 1500 kDa). HA samples were dissolved at a concentration, based on dry weight reported by the manufacturer, of 1 mg/mL in sterile PBS and stored at 4°C. An HA concentration of 0.5 mg/mL in PBS allowed CD data to be obtained to approximately 200 nm using a 0.1 cm optical cell. An average residue weight of 401 g/mol for the repeating disaccharide of HA sodium salt was used to calculate molar ellipticity for the spectrum of HA alone. Mixtures of HA with peptide were prepared by mixing equal volumes of HA at 1 mg/mL and peptide at 0.4 mg/mL. The samples were left at room temperature for 2 h before the CD spectra measurements.

The CD spectra were deconvoluted for secondary structure analyses of samples using BeStSel.^51^

### Peptide structure prediction

Peptide structures were modeled using AlphaFold2.^37^ Amino acid sequences of the peptides were entered through the ColabFOLD notebook platform (version 1.5.5) (https://colab.research.google.com/github/sokrypton/ColabFold/blob/main/AlphaFold2.ipynb) (accessed in February 2024).^52^ The ColabFold notebook was run with the default settings; the ‘num_relax’ and ‘template_mode’ parameters were set to 0 and ‘none’, respectively. For the multiple sequence alignment (MSA) required for the AlphaFold modeling, ‘mmseqs. 2_uniref_env’ and ‘unpaired_paired’ were selected for the ‘msa_mode’ and ‘pair_mode,’ respectively. In the advanced setting options, the ‘model_type’ was set to ‘auto,’ the ‘num_recycles’ to 3 (48 recycles were used for the 32 aa RHAMM peptide) ‘recycle_early_stop_tolerance’ to ‘auto,’ ‘relax_max_iterations’ to 200, and the ‘pairing_strategy’ to ‘greedy.’ In the sample settings section, the ‘max_msa’ was set to ‘auto’, and the ‘num_seeds’ was set to 1. Out of the top five structure models generated for each sequence by AlphaFold2, only those with predicted Local Distance Different Test (pLDDT) scores, often referred to as confidence scores, exceeding 70 were considered. This range of pLDDT scores indicates a ‘high’ to ‘very high’ level of confidence in structure prediction.^38^ Conversely, structure models with pLDDT scores lower than 70, indicating ‘low’ to ‘very low’ confidence,^38^ were not selected for consideration. The outputs from AlphaFold‐based structure models were downloaded in the PDB format and visualized using PyMOL 3.0 (The PyMOL Molecular Graphics System, Version 3.0 Schrödinger, LLC) and the Mol* 3D viewer.^53^

### TANGO analysis

The aggregation propensities for the peptides were calculated using a sequence‐based amyloid aggregation predictor, TANGO.^54^ For this analysis, amino acid sequences of the peptides were entered through the TANGO web server (http://tango.crg.es/), which then returned the calculated aggregation propensity scores that were used directly.

## RESULTS

### The RHAMM‐competitive P15‐1 peptide adopts irregular conformations in solution

**Figure 2:**
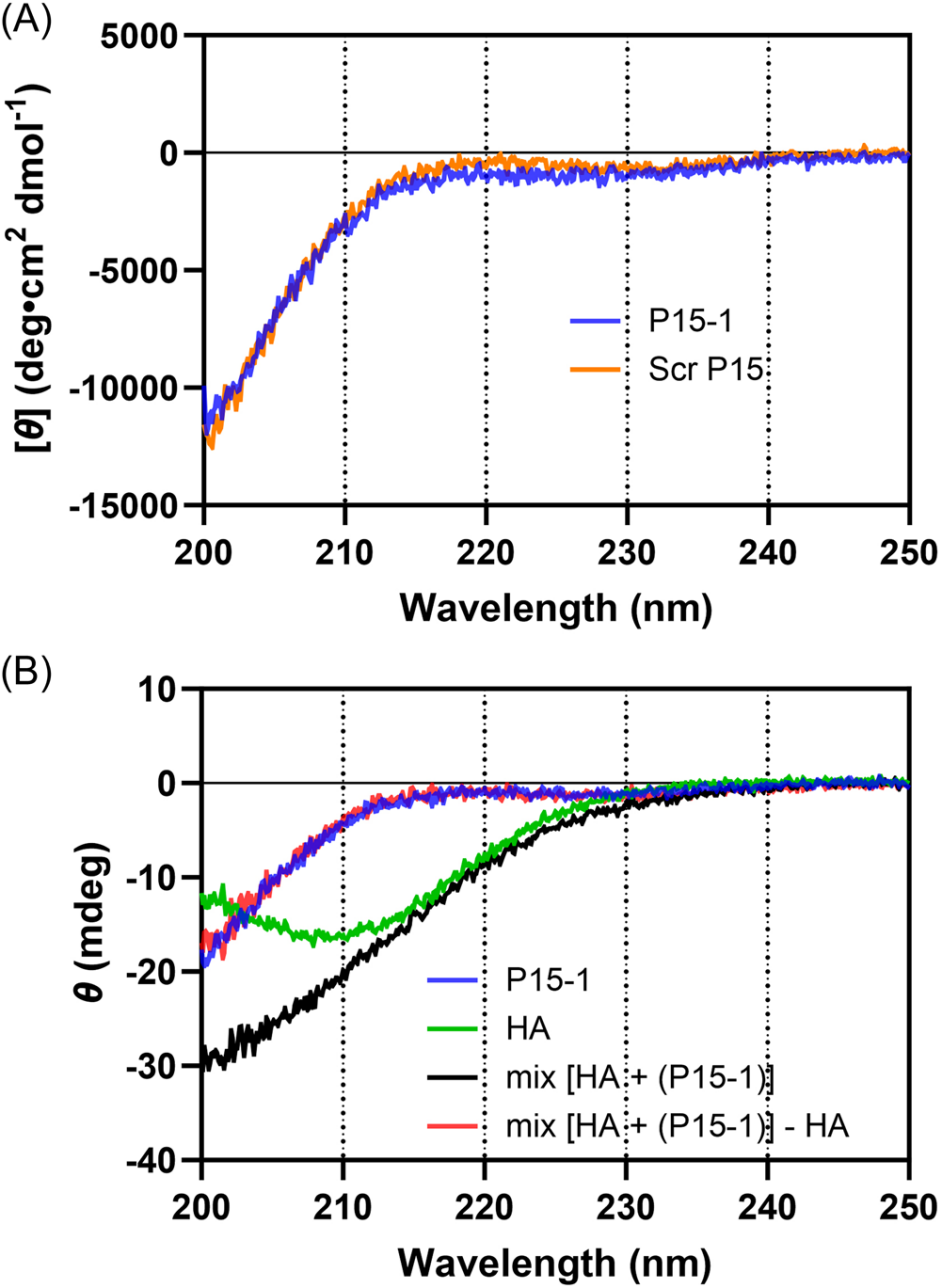
Circular dichroism spectra of P15‐1 and ScrP15 peptide in phosphate buffered saline (PBS), and P15‐1 in admixture with 50 kDa hyaluronan (HA) in PBS, show the peptide conformations are primarily disordered in both cases. (A) Peptides at 0.2 mg/mL in PBS. (B) P15‐1 at 0.2 mg/mL and HA at 0.5 mg/mL in PBS.

The CD spectra of P15‐1 (STMMSRSHKTRSHHV) and scrambled P15‐1 (ScrP15; HKSVSRHTSMRHSTM) in phosphate‐buffered saline (PBS) recorded at 25°C were closely similar and were dominated by negative ellipticity from one or more bands centered below 200 nm (Figure 2A). Based on this spectral signature, both 15‐mer peptides appear to be in primarily disordered random coil forms. Deconvolution of the P15‐1 CD spectrum using BeStSel^51^ suggests a population of approximately 51% irregular structures, along with 29% beta‐sheet structures and 20% turns. This is in contrast to the alpha‐helical conformations previously observed for ca. 60 aa HA‐binding domain fragments of RHAMM protein.^40^, ^41^

The CD spectra of P15‐1 and ScrP15 peptides mixed with HA in solution were also analyzed, to determine if the peptides adopted more ordered (especially alpha helical) conformations when bound to, or molecularly crowded by, HA. The CD spectrum of HA alone (Figure 2B) showed a negative band centered near 209 nm with a molar ellipticity of −1.3 × 10^4 ^deg cm^2^ dmol^−1^, calculated from the ellipticity data shown in the figure, using the molar HA disaccharide concentration of 1.25 × 10^−3^ M ( = 1.25 × 10^−5^ dmol cm^−3^) and the path length of 0.1 cm, in agreement with a previous report.^55^ The spectra for mixtures of HA with P15‐1 (Figure 2B) or ScrP15 (Supporting Information: Figure S1) are also shown in raw data units of ellipticity, because both components contribute to the CD signal, so that no single concentration would be appropriate to use to scale the data. The CD spectra for the mixtures were found to be indistinguishable from additive spectra of the HA and peptide components, and subtraction of the HA component from the spectra for the mixtures yielded spectra for the peptides that were unchanged from the free peptide spectra. Thus, no evidence for an increase in ordered secondary structure of the peptides was found. This result is independent of the HA molecular weight, from 50 to 500 to 1500 kDa (Supporting Information: Figure S2).

### AlphaFold2 predictions for peptides show that sequence and conformation combine to determine spacing of basic amino acids

**Figure 3:**
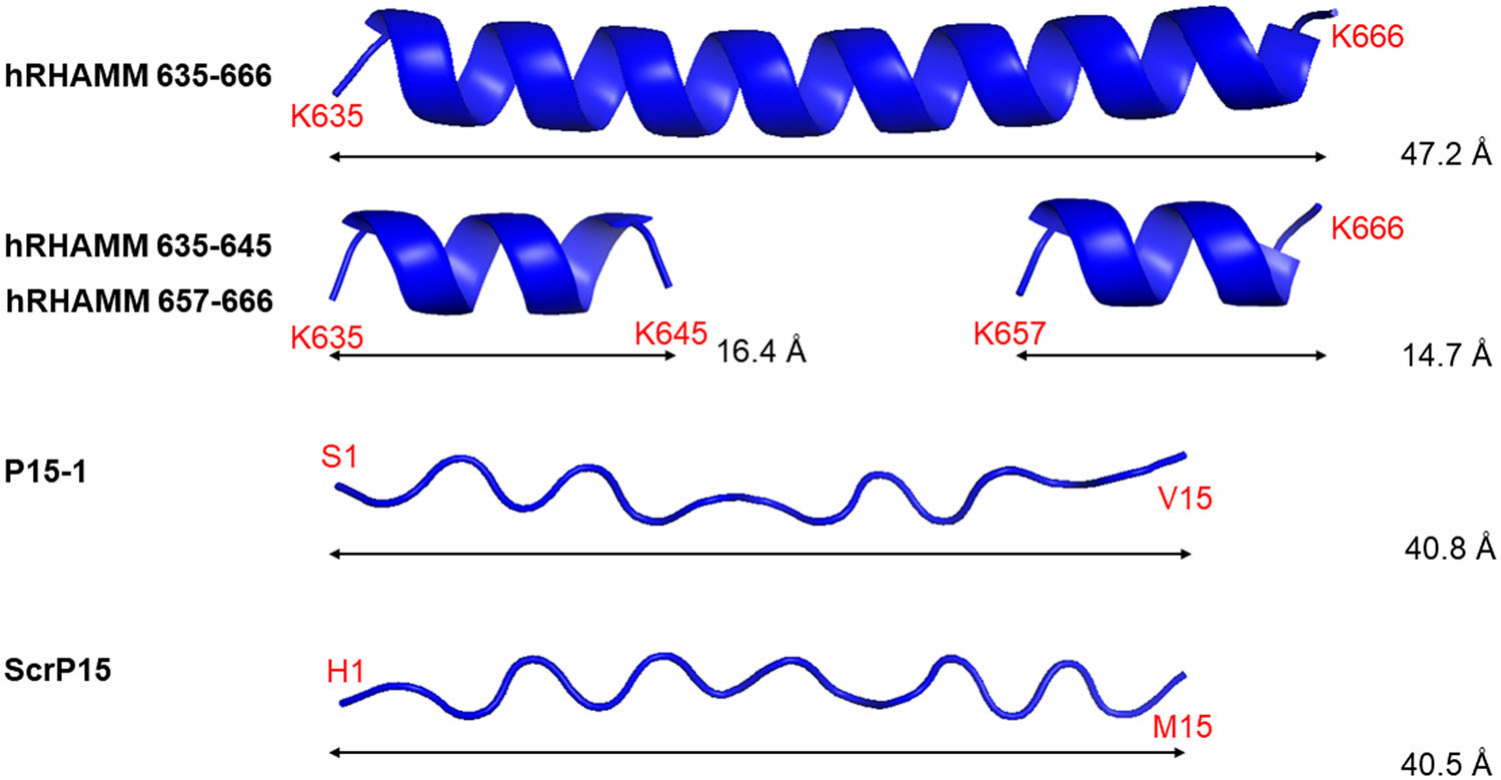
Predicted backbone conformations of hyaluronan (HA)‐binding receptor for hyaluronan‐mediated motility (RHAMM) peptides, the RHAMM‐competitive P15‐1, and a scrambled version of P15‐1, modeled using AlphaFold2.^37^, ^38^ The RHAMM peptides are all predicted to adopt alpha‐helical conformations, while the RHAMM‐competitive P15‐1 and its scrambled version are predicted to adopt extended irregular backbone conformations. Structures depicted are the top‐ranked models, with pLDDT scores of 93.1, 84.4, 89.3, 78.2, and 77.4 (top to bottom, respectively), corresponding to very high (>90) and high (70–90) confidence levels. All structures were visualized with PyMOL 3.0 software.

The experimentally determined conformational difference between the 15‐mer P15‐1 and the ca. 60 aa RHAMM protein segments in solution might reflect their inherent differences in conformational preference, which may be revealed by predictive modeling approaches. For example, using AlphaFold2 for structure modeling, the human RHAMM 32 aa HA‐binding sequence from K635 to K666, and the two critical BX_7_B‐containing sequences of K635 to K645 and K657 to K666, are all predicted with high confidence to be primarily alpha‐helical (top‐ranked backbone conformations are shown in Figure 3, and full results for models with pLDDT confidence scores above 70 in Supporting Information: Figures S3–S5). In contrast, both P15‐1 and ScrP15 peptides are predicted with high confidence to adopt extended irregular structures (Figure 3; Supporting Information: Figures S6 and S7). The predicted alpha‐helical conformations for RHAMM peptides and irregular extended conformations for the P15‐1/ScrP15 peptides are in good agreement with experimental CD results from solution studies. The differences in aa sequence result in fundamentally different conformational preferences.

**Figure 4:**
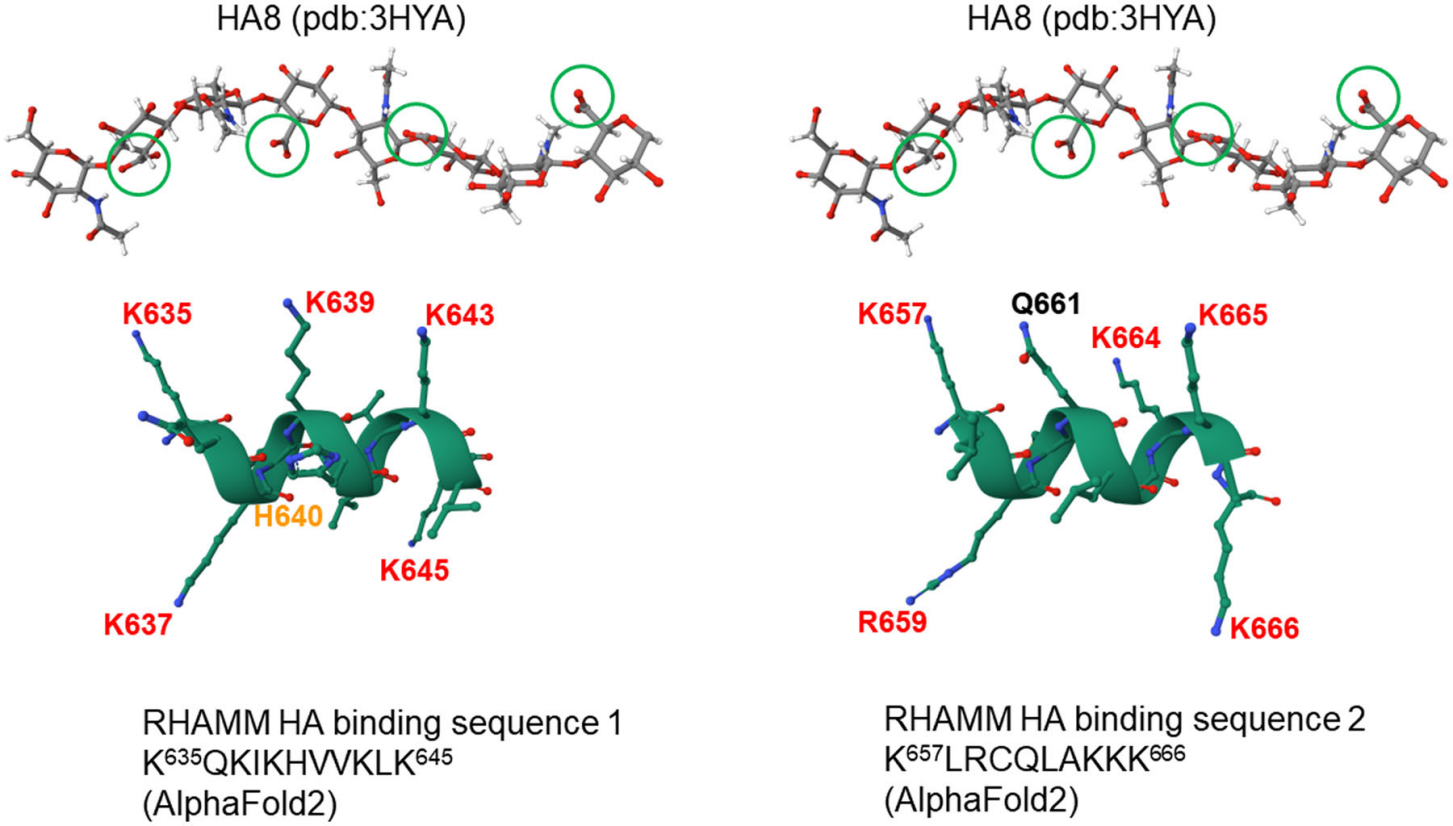
The top‐ranked models for the two hyaluronan (HA) binding sequences in human receptor for hyaluronan‐mediated motility (RHAMM) show the potential for matched spacing of basic amino acid side chains in the BX_7_B motif with three consecutive carboxylates in HA. Peptide models are calculated using AlphaFold2.^37^, ^38^ The basic R and K residues are labeled in red (orange for H). The HA octasaccharide (HA8) structure, determined by X‐ray fiber diffraction analysis,^56^ was downloaded from the RCSB PDB (rcsb.org), PDB ID 3HYA (https://doi.org/10.2210/pdb3HYA/pdb). All structures were visualized with Mol* software.^53^ The four carboxylate groups in HA8 are shown enclosed by green circles.

To better understand the relationship between peptide sequence, conformation, and HA binding, the top‐ranked predicted structures of peptides including aa side chains were scaled based on the distance between N‐ and C‐terminal carbon atoms as shown in Figure 3, and then visually aligned against the structure of a 33.9 Å long octasaccharide unit of sodium HA (HA8) determined by X‐ray fiber diffraction analysis (Figure 4),^56^ downloaded from the RCSB PDB (rcsb.org), PDB ID 3HYA (https://doi.org/10.2210/pdb3HYA/pdb).

In human RHAMM, HA binding sequence 1 (K635–K645) and sequence 2 (K657–K666) each present three suitably spaced and oriented K side chains within a BX_7_B motif to interact with three consecutive carboxylate groups of HA (Figure 4). The combination of the BX_7_B sequence motif with the alpha‐helical conformation dictates the favorable spacing of the basic aa side chains. It is interesting to note that each RHAMM peptide also displays a second set of K and/or R groups facing the opposite side of the helix, which may contribute to the overall affinity of HA binding by increasing the linear charge density.

**Figure 5:**
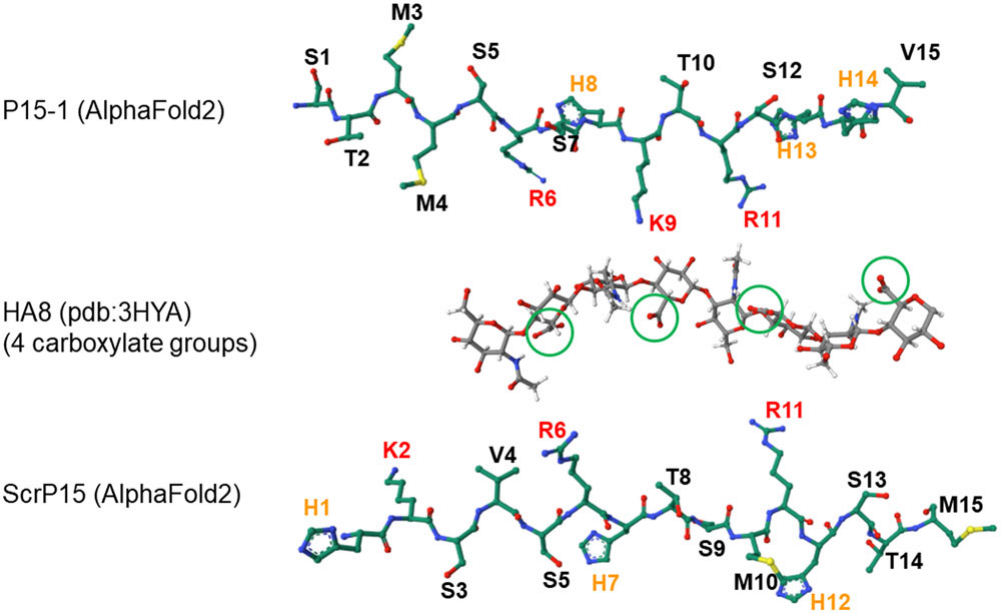
The top‐ranked conformational model predicted for peptide P15‐1 shows an irregular backbone with the potential for matched spacing of basic amino acid side chains with three consecutive carboxylates in hyaluronan (HA) while ScrP15 matches two nonconsecutive HA carboxylates. Peptide models are calculated using AlphaFold2.^37^, ^38^ The basic R and K residues are labeled in red (H in orange). The HA octasaccharide (HA8) structure, determined by X‐ray fiber diffraction analysis,^56^ was downloaded from the RCSB PDB (rcsb.org), PDB ID 3HYA (https://doi.org/10.2210/pdb3HYA/pdb). All structures were visualized with Mol* software.^53^ The four carboxylate groups in HA8 are shown enclosed by green circles.

The P15‐1 and ScrP15 peptides, in their predicted extended irregular conformations, can also present basic aa side chains for electrostatic interaction with HA (Figure 5). In the extended conformation predicted by AlphaFold2, P15‐1 shows clustering of basic side chains that causes all six (two R, one K, three H) to fall within a distance corresponding to the spacing of the four carboxylates of HA8, and the spacing of the R6, K9, and R11 side chains closely approximates that of three consecutive HA carboxylates. The basic residues of ScrP15 are more evenly spread out over the full length of that peptide. The spacing of side chains for R6 and R11 match the spacing between two HA carboxylates, but these are nonconsecutive. This comparison of the predicted P15‐1 and ScrP15 structures suggests an explanation for the significant difference in HA binding affinity between the two peptides.

**Figure 6:**
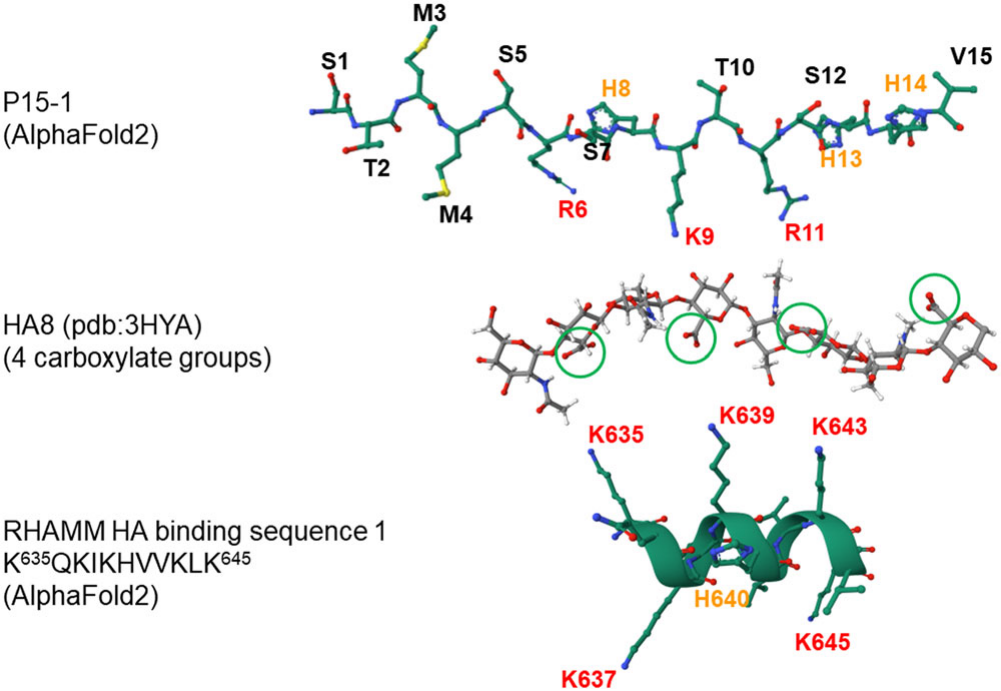
The top‐ranked conformational models predicted for the hyaluronan (HA) binding sequence 1 of human receptor for hyaluronan‐mediated motility (RHAMM) and peptide P15‐1 show similarly matched spacing of basic amino acid residues with HA carboxylates can be achieved by the irregular backbone of P15‐1 or the alpha‐helical backbone of RHAMM. Peptide models are calculated using AlphaFold2.^37^, ^38^ The basic R and K residues are labeled in red (H in orange). The HA octasaccharide (HA8) structure, determined by X‐ray fiber diffraction analysis,^56^ was downloaded from the RCSB PDB (rcsb.org), PDB ID 3HYA (https://doi.org/10.2210/pdb3HYA/pdb). All structures were visualized with Mol* software.^53^ The four carboxylate groups in HA8 are shown enclosed by green circles.

The ability of P15‐1, with its extended irregular conformation, to effectively compete with the alpha‐helical RHAMM sequence for HA binding is illuminated by direct comparison of basic aa spacing. Figure 6 shows that both P15‐1 and RHAMM HA binding sequence 1 display R or K basic aa side chains in suitable spacing and orientation for electrostatic interaction with three consecutive HA carboxylates. The human RHAMM HA binding sequence 1, K635‐K645 contains six basic aa in two overlapping BX_7_B motifs, in a compact alpha‐helical conformation. Depending on the rotation of the peptide relative to HA, three residues of a single BX_7_B motif (e.g., K635, K639, and K643) can spatially match three HA carboxylates. In P15‐1, the three strongly basic aa R6, K9, and R11 represent a shorter primary sequence between basic residues that is compensated by a more extended irregular backbone conformation, resulting in spatial matching with three consecutive HA carboxylates. Because R residues have stronger affinity for HA carboxylates than K residues,^57^ the R6, K9, R11 pattern in P15‐1 increases its ability to compete with K residues in RHAMM.

It may be of interest to apply the conformation‐predictive AI‐based AlphaFold2 algorithm to examine the structures of other known HA‐binding peptides, to guide discovery and/or design of new peptides with therapeutic potential. For example, the rationally designed peptides YKQKIKHVVKLK (based on the RHAMM K635‐K645 sequence with addition of a Y residue at the N terminal, and here called YRHAMM1) and RGGGRGRRR (containing a BX_7_B motif, and here called RG peptide) have been shown to compete with RHAMM for HA binding, and to have therapeutic potential.^43^ We have analyzed these two peptides by AlphaFold2 (Supporting Information: Figures S8–S10). The YRHAMM1 peptide favors an alpha‐helical conformation, essentially unchanged from the RHAMM sequence, with three K residues suitably spaced for interaction with HA carboxylates. The RG peptide is predicted to adopt a more irregular extended conformation, with a cluster of four closely spaced R residues (R5, R7, R8, R9), suggesting the potential for favorable electrostatic interaction with 2‐3 HA carboxylates. A very different type of HA binding peptide discovered by unbiased phage display technology, Pep‐1 (GAHWQFNALTVR), has also been shown to have therapeutic potential.^58^ The peptide sequence has two basic aa but does not contain a BX_7_B motif and is not RHAMM‐mimetic. It has multiple hydrophobic and aromatic aa as well as uncharged polar aa bearing amide or hydroxyl groups. Using AlphaFold2, Pep‐1 conformation is predicted to be irregular and extended, with approximately the same length as an octamer of HA (Supporting Information: Figures S8, S11), and offering the potential for multiple types of favorable interactions such as electrostatic, hydrogen bonding, and CH‐π interactions, common in the carbohydrate‐binding domains of proteins.^59^

### Analysis of aggregation potential of the RHAMM‐competitive peptide P15‐1

An additional consideration of interest with respect to therapeutic peptides like P15‐1 is the propensity for potentially problematic aggregation in solution. The aggregation propensities of P15‐1 and scrP15 were assessed using a sequence‐based aggregation predictor, TANGO.^54^ TANGO considers physicochemical parameters (e.g., hydrophobicity and charge) of amino acids and competition between different conformational states for the aggregation prediction.^54^ Our TANGO‐based analysis shows that both P15‐1 and scrP15 have negligible aggregation propensities (aggregation score ~ 0).

## DISCUSSION

RHAMM protein has two BX_7_B‐containing domains located closely together near the C‐terminal end of the protein, and this is the site of HA binding. Using the AlphaFold algorithm the HA‐binding region of RHAMM protein is predicted to adopt a primarily alpha‐helical conformation (Figure 1), consistent with the alpha‐helical structure experimentally determined by CD spectroscopy for 60‐62 aa long HA‐binding fragments of murine RHAMM.^40^, ^41^ The alpha‐helical conformation dictates the spacing and orientation of basic side chains and, coupled with the BX_7_B sequence, results in a suitable pattern for interaction with carboxylate groups on every other sugar residue in HA. For short RHAMM peptides with BX_7_B motifs, chain conformations have not previously been investigated experimentally, but here are predicted by the AlphaFold2 method to favor alpha helices (Figures 3, 4, S4‐S6). The importance of the BX_7_B motif for HA binding by RHAMM is intrinsically linked to the alpha‐helical peptide backbone conformation.

While maintaining a predominantly helical conformation, an alternative folded 3 helix bundle tertiary structure has been proposed for a 60‐aa murine RHAMM sequence (corresponding to human RHAMM residues 621‐680), in which the first BX_7_B motif lies in the first helix, and the second BX_7_B motif lies in the second helix, based on NMR data and modeling with an early database of protein sequences and corresponding folded structures.^41^ The predictive scheme used here, based on the AlphaFold2 algorithm and a larger database of known protein structures, does not suggest any break in alpha helix requiring the formation of disordered loops within the 32‐aa sequence containing both BX_7_B HA‐binding domains. While further experimental study is warranted, the BX_7_B motifs are alpha helical in either model, and present the same spatial pattern of basic aa residues for interaction with HA.

In the present study, we have undertaken a comparative experimental investigation of the solution conformations of the RHAMM‐competitive P15‐1 peptide and its scrambled version ScrP15, as well as conformational predictions for those peptides using the AlphaFold2 simulation method. P15‐1 was discovered using an unbiased phage display peptide library screened for peptides binding to HA but not chondroitin sulfate, and to disrupt HA‐RHAMM binding.^29^ It contains a BX_7_B motif, but with H replacing R or K for the second basic residue, which would severely weaken its affinity for HA at neutral pH. Instead, it has three closely spaced R/K residues within a six aa sequence.

Our experimental CD results indicate that, at neutral pH, both P15‐1 and ScrP15 are mostly irregularly structured with beta sheets as a minor structural component. In addition, our computational modeling of P15‐1 and ScrP15 (Figures 3, 5; Supporting Information: Figures S7, S8) suggests that both peptides have extended irregular conformations. Together, these results indicate that a stable alpha‐helical conformation is not required for binding between HA and P15‐1 peptide or ScrP15. The peptide structures predicted by AlphaFold2 suggest that the extended chain conformation of P15‐1 can provide suitable spacing of three R/K basic residues in a short six aa sequence for sufficiently strong interaction with HA carboxylates to compete with RHAMM (Figure 6).

The question of whether or not the conformation of RHAMM peptide mimetics is pH dependent is an interesting consideration, especially with respect to understanding the role of histidine in HA binding. The pH of the microenvironment during injury responses is tissue context dependent but generally ranges from 6 to 8.9.^60^ This pH range did not affect RHAMM:HA interactions in preliminary experiments. In contrast to tissue injury, tumor microenvironments are acidic and have been reported to range from 5.6 to 6.8 due to tumor cell glycolysis, hypoxia and poor blood perfusion.^61^ We have not measured the effects of these pH ranges on RHAMM protein or RHAMM mimetic peptides but this is a very interesting future avenue to investigate experimentally. The question cannot be addressed by AlphaFold2 modeling, as it is based on learning from PDB structures (which are mostly determined for the native state at neutral pH).

Conformationally disordered structural features are commonly shared with monomeric forms of many aggregation‐prone amyloid fibril‐forming proteins.^62^, ^63^ Interestingly, the CD spectra of P15‐1 and its scrambled version are similar to those previously taken for structurally disordered monomeric forms of amyloid proteins, such as beta‐amyloid and alpha‐synuclein, with a weak minimum at ∼230 nm, a plateau at ∼220 nm, and a steady increase of negative ellipticity toward shorter wavelengths from ∼215 to 200 nm.^64^ In a recent report, some longer (23–27 amino acids) peptides containing multiple BX_7_B motifs were observed to aggregate and form gels or networks of fibrils at high peptide concentrations.^65^ In contrast, P15‐1 is predicted to have negligible propensity to form amyloid fibrils, when examined by an amyloid aggregation predictor, TANGO,^54^ and we have not observed any evidence for aggregation of P15‐1.

As a potential therapeutic approach to treatment of multiple diseases and injuries involving inflammation and fibrosis, peptides that can modulate the extracellular interactions among HA, CD44 and RHAMM show promise. The clustering of CD44 when bound to high molecular weight HA is the homeostatic state. Under inflammatory conditions, de‐clustering of CD44 and its subsequent proteolytic cleavage and/or interactions with other proteins such as growth factor receptors are modulated by HA fragmentation caused by enzymatic and reactive oxygen/nitrogen species. At the same time, export of the cytoplasmic protein RHAMM to the extracellular matrix results in its interaction with HA and CD44. This new signaling complex of HA fragments with CD44 and RHAMM is associated with increased cell motility and pro‐inflammatory, pro‐fibrotic changes in gene expression and protein levels or activation.^22^ While the constitutive expression of CD44 makes it difficult to target therapeutically, orthogonal targeting of a partner protein, extracellular RHAMM, which is released with injury or disease, is very attractive. Thus, a peptide such as P15‐1 that binds HA and disrupts HA‐RHAMM interaction may offer an approach to minimize the pro‐inflammatory signaling. Future exploration of this potential mechanism of action and testing of P15‐1 in preclinical and clinical settings may help optimize potential therapeutic approaches for improved tissue remodeling and repair following injury or disease.

## AUTHOR CONTRIBUTIONS

**Mehmet Emre Erkanli**: Methodology; investigation; data curation; writing—review and editing; visualization. **Ted Keunsil Kang**: Methodology; investigation. **Thorsten Kirsch**: Conceptualization; resources; writing—review and editing; funding acquisition. **Eva A. Turley**: Conceptualization; writing—review and editing. **Jin Ryoun Kim**: Conceptualization; methodology; formal analysis; investigation; resources; data curation; writing—original draft; writing—review and editing; visualization; supervision; project administration. **Mary K. Cowman**: Conceptualization; methodology; formal analysis; investigation; resources; data curation; writing—original draft; writing—review and editing; visualization; supervision; project administration; funding acquisition.

## CONFLICT OF INTEREST STATEMENT

Mary Cowman, Thorsten Kirsc,h and Eva Turley are listed as inventors on US patent 10,449,229 B2. The rest of the authors declare no conflict of interest. The sponsors had no role in the design, execution, interpretation, or writing of the study.

## ETHICS STATEMENT

The authors have nothing to report.

## PERMISSION TO REPRODUCE MATERIALS

Figure 1 is adapted with permission [Cowman and Turley, 2023, Proteoglycan Research 1(2): p. e4; https://doi.org/10.1002/pgr2.4]. Copyright 2023 The Authors. Proteoglycan Research is published by Wiley Periodicals LLC.

## Supporting information

Supporting information.

## Data Availability

The data that support the findings of this study are available from the corresponding author upon reasonable request.
