# Supplementary material for: The spatial separation of basic amino acids is similar in RHAMM and hyaluronan binding peptide P15‐1 despite different sequences and conformations
Source: Proteoglycan Res. 2024 Sep 10;2(3):e70001. doi: 10.1002/pgr2.70001 (PMC11404675; doi:10.1002/pgr2.70001)
Supplement: Supplementary file 1 — Supporting information. [file PGR2-2-e70001-s001.pdf]

Supporting Information

The spatial separation of basic amino acids is similar in RHAMM and hyaluronan binding peptide P15-1 despite different sequences and conformations

Mehmet Emre Erkanli, Ted Keunsil Kang, Thorsten Kirsch, Eva A. Turley, Jin Ryoun Kim, and Mary K. Cowman

Methods

Circular Dichroism (CD) spectra were recorded using a Jasco J-810 spectropolarimeter (Easton, MD, USA), at 25 ^{o}C. Spectroscopic parameters and sample concentrations were optimized to allow data acquisition over a wavelength range from 250 nm to 200 nm, using a spectral bandwidth of 1 nm, response time 1 s, and scan rate 10 nm/min, allowing 6 response periods per unit of spectral bandwidth. Spectral data were averaged from 5 scans. The quartz optical cell path length was 0.1 cm. The average residue weight per amino acid of 119 g/mol was used to calculate molar ellipticity. No smoothing was applied to the data. A baseline control spectrum of PBS was subtracted from each sample spectrum.

Peptide structures were modeled using AlphaFold2 [Jumper et al. 2021]. Amino acid sequences of the peptides were entered through the ColabFOLD notebook platform (version 1.5.5) (https://colab.research.google.com/github/sokrypton/ColabFold/blob/main/AlphaFold2.ipynb) [Mirdita et al. 2022]. Out of the top five structure models generated for each sequence by AlphaFold2, only those with predicted Local Distance Different Test (pLDDT) scores, often referred to as confidence scores, exceeding 70 were considered. This range of pLDDT scores indicates a 'high' to 'very high' level of confidence in structure prediction [Varadi et al. 2022]. Conversely, structure models with pLDDT scores lower than 70, indicating 'low' to 'very low' confidence, were not selected for consideration. The outputs from AlphaFold-based structure models were downloaded in the PDB format and visualized using PyMOL 3.0 (The PyMOL Molecular Graphics System, Version 3.0 Schrödinger, LLC).

References

Jumper, J., R. Evans, A. Pritzel, T. Green, M. Figurnov, O. Ronneberger, K. Tunyasuvunakool, R. Bates, A. Zidek, A. Potapenko, A. Bridgland, C. Meyer, S. A. A. Kohl, A. J. Ballard, A. Cowie, B. Romera-Paredes, S. Nikolov, R. Jain, J. Adler, T. Back, S. Petersen, D. Reiman, E. Clancy, M. Zielinski, M. Steinegger, M. Pacholska, T. Berghammer, S. Bodenstein, D. Silver, O. Vinyals, A. W. Senior, K. Kavukcuoglu, P. Kohli and D. Hassabis (2021). "Highly accurate protein structure prediction with AlphaFold." Nature 596(7873): 583-589.

Mirdita, M., K. Schutze, Y. Moriwaki, L. Heo, S. Ovchinnikov and M. Steinegger (2022). "ColabFold: making protein folding accessible to all." Nat Methods 19(6): 679-682.

Varadi, M., S. Anyango, M. Deshpande, S. Nair, C. Natassia, G. Yordanova, D. Yuan, O. Stroe, G. Wood, A. Laydon, A. Zidek, T. Green, K. Tunyasuvunakool, S. Petersen, J. Jumper, E. Clancy, R. Green, A. Vora, M. Lutfi, M. Figurnov, A. Cowie, N. Hobbs, P. Kohli, G. Kleywegt, E. Birney, D. Hassabis and S. Velankar (2022). "AlphaFold Protein Structure Database: massively expanding the structural coverage of protein-sequence space with high-accuracy models." Nucleic Acids Res 50(D1): D439-D444.

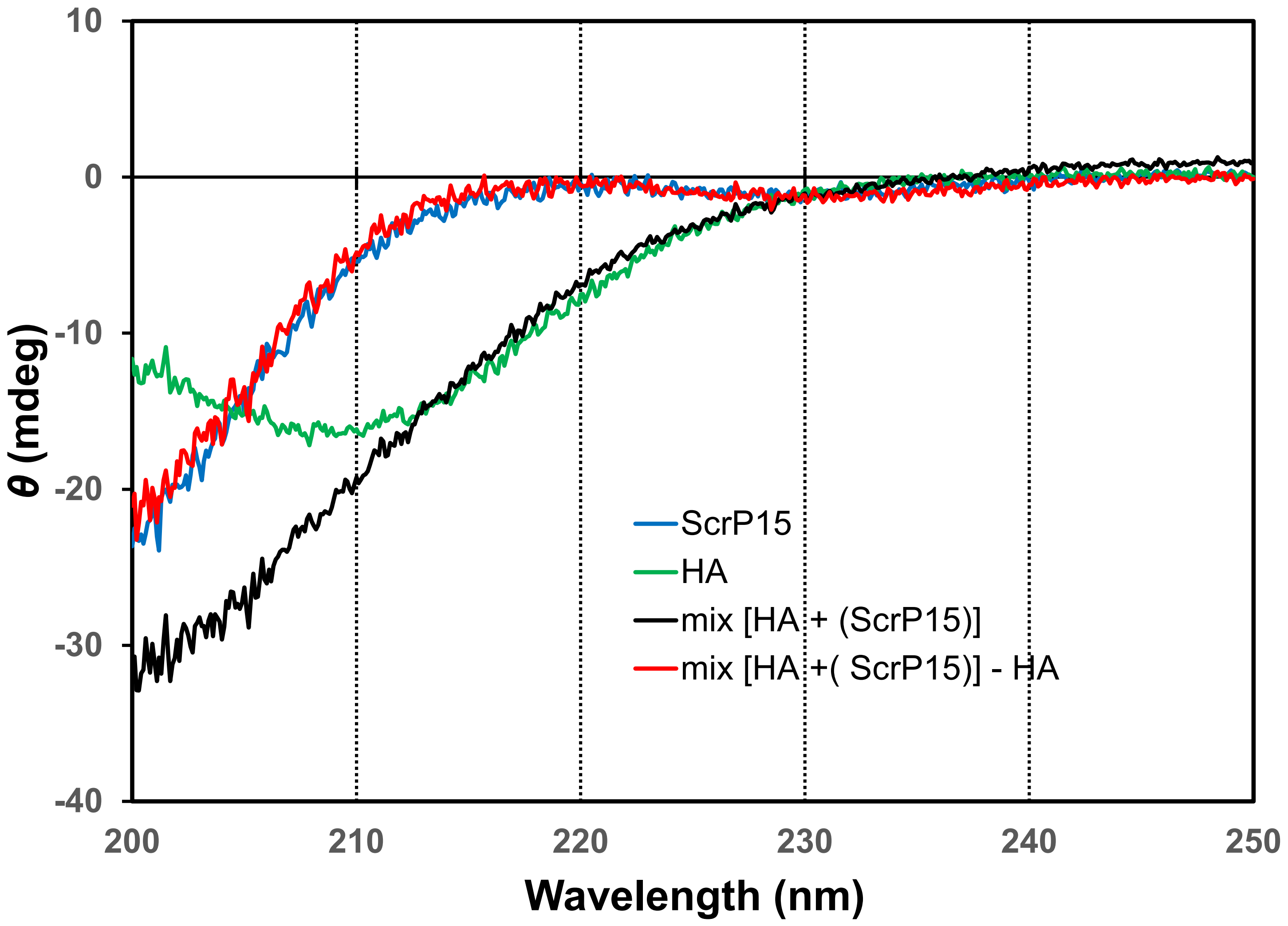

Figure S1. Circular dichroism spectra of ScrP15 peptide in phosphate buffered saline (PBS), and in admixture with HA in PBS, show the peptide conformation is unchanged by HA. ScrP15 at 0.2 mg/mL and HA at 0.5 mg/mL in PBS.

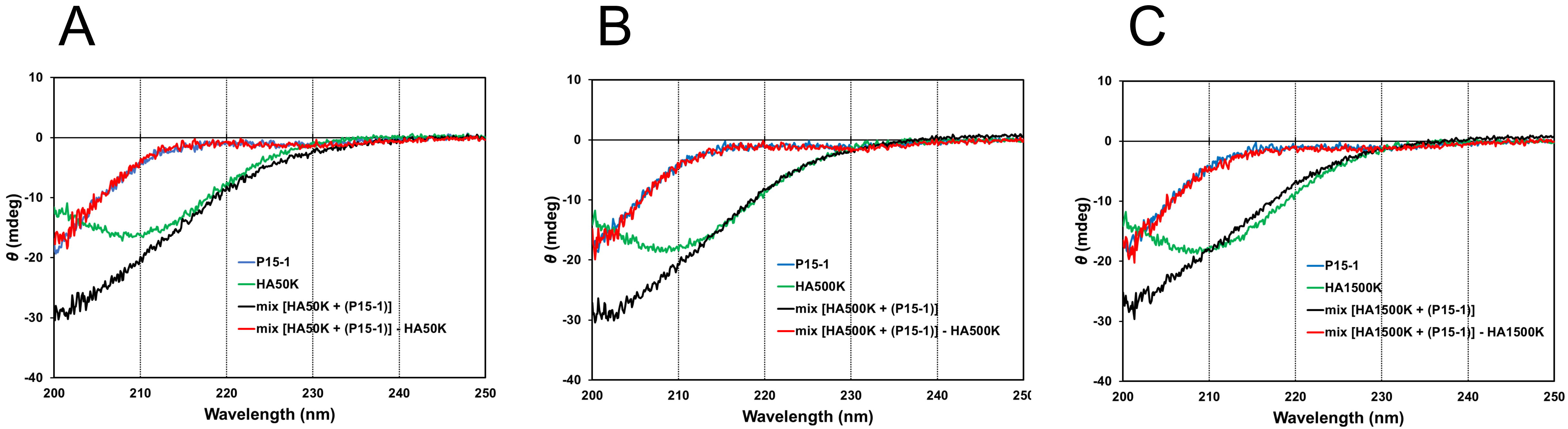

Figure S2. Circular dichroism spectra of P15-1 peptide in phosphate buffered saline (PBS), and in admixture with HA in PBS, as a function of HA molecular weight, show the peptide conformation remains unchanged by HA in all cases. P15-1 at 0.2 mg/mL and HA at 0.5 mg/mL in PBS.
HA molecular weight was A) 50 kDa, B) 500 kDa, C) 1500 kDa.

hRHAMM K635-K666, AlphaFold2

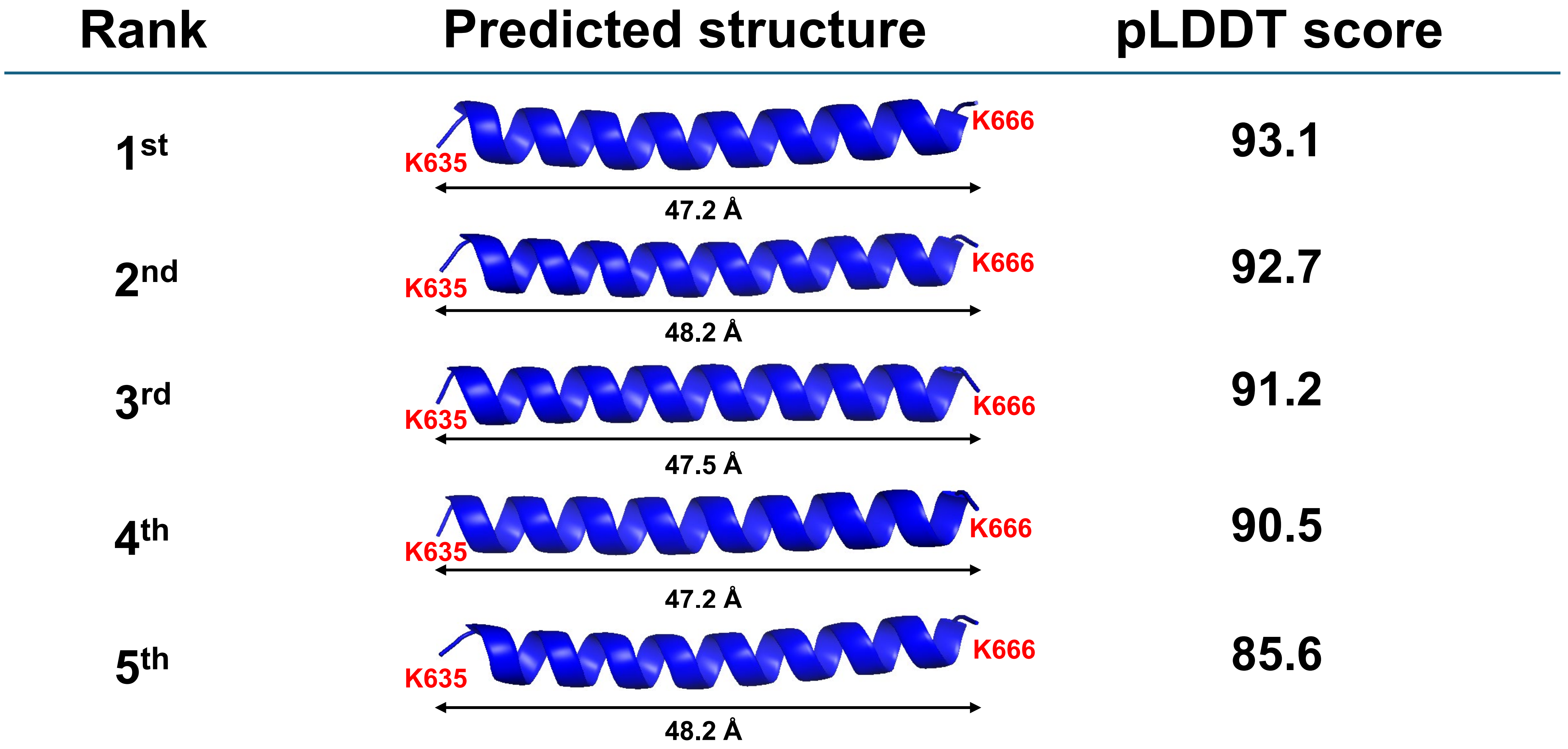

Figure S3. The top models for human RHAMM (hRHAMM) K635-K666 predicted by AlphaFold2 (Jumper et al., 2021; Varadi et al. 2022) along with their pLDDT scores are presented. Only the models with pLDDT scores exceeding 70 are shown. The corresponding lengths of the predicted structures are displayed under the models.

hRHAMM K635-K645, AlphaFold2

Rank	Predicted structure	pLDDT score
1st	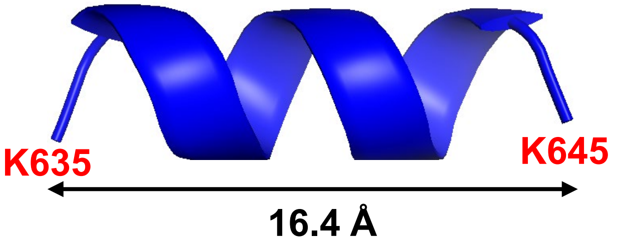	84.4
2nd	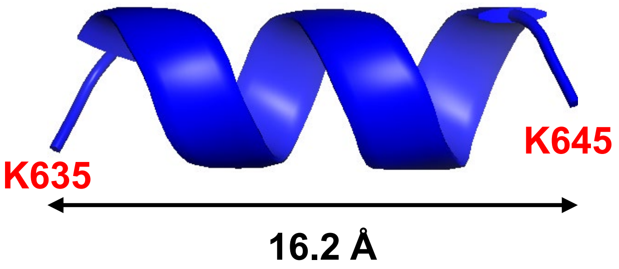	78.4
3rd	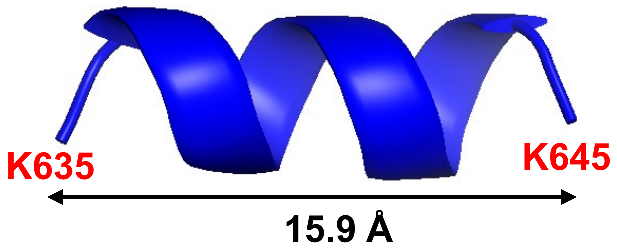	76.8

Figure S4. The top models for human RHAMM (hRHAMM) K635-K645 predicted by AlphaFold2 (Jumper et al., 2021; Varadi et al. 2022) along with their pLDDT scores are presented. Only the models with pLDDT scores exceeding 70 are shown. The corresponding lengths of the predicted structures are displayed under the models.

hRHAMM K657-K666, AlphaFold2

Rank	Predicted structure	pLDDT score
1st	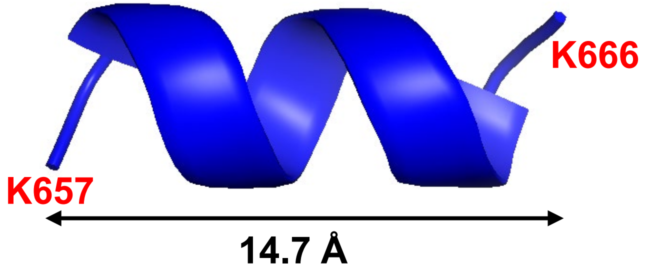	89.3
2nd	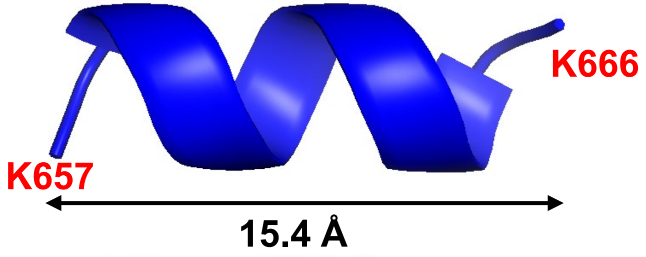	87.4
3rd	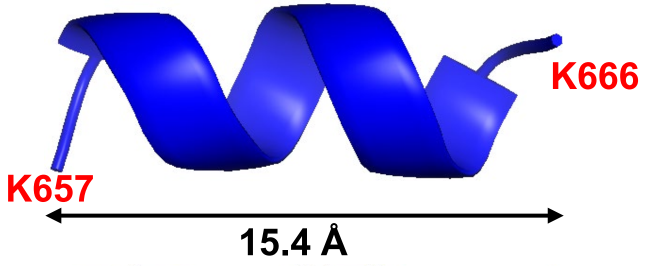	82.1
4th	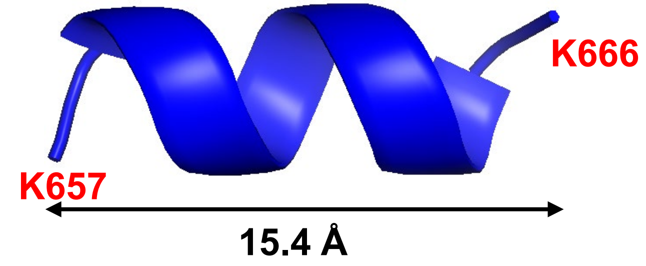	77.4

Figure S5. The top models for human RHAMM (hRHAMM) K657-K666 predicted by AlphaFold2 along with their pLDDT scores are presented. Only the models with pLDDT scores exceeding 70 are shown. The corresponding lengths of the predicted structures are displayed under the models.

P15-1, AlphaFold2

Rank	Predicted structure	pLDDT score
1st	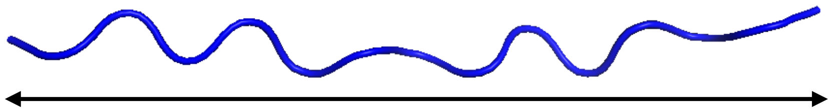 40.8 Å	78.2
2nd	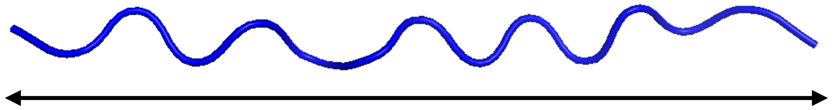 41.3 Å	73.4
3rd	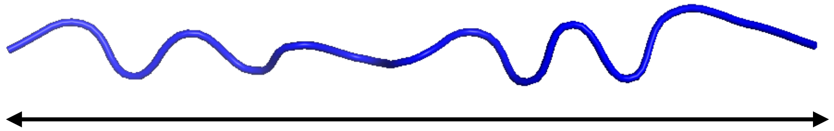 39.9 Å	70.3

Figure S6. The top models for P15-1 predicted by AlphaFold2 along with their pLDDT scores are presented. Only the models with pLDDT scores exceeding 70 are shown. The corresponding lengths of the predicted structures are displayed under the models.

ScrP15, AlphaFold2

Rank	Predicted structure	pLDDT score
1st	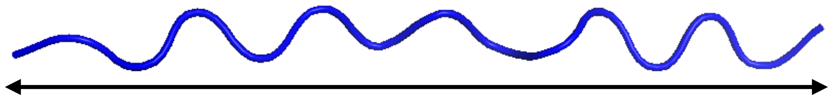 40.5 Å	77.4
2nd	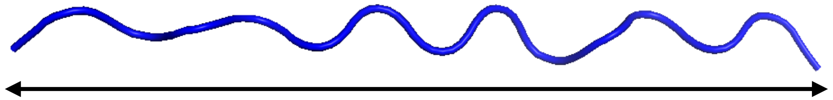 40.5 Å	75.9
3rd	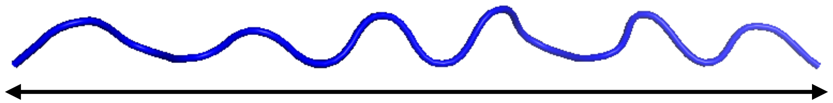 42.1 Å	71.6

Figure S7. The top models for ScrP15 predicted by AlphaFold2 along with their pLDDT scores are presented. Only the models with pLDDT scores exceeding 70 are shown. The corresponding lengths of the predicted structures are displayed under the models.

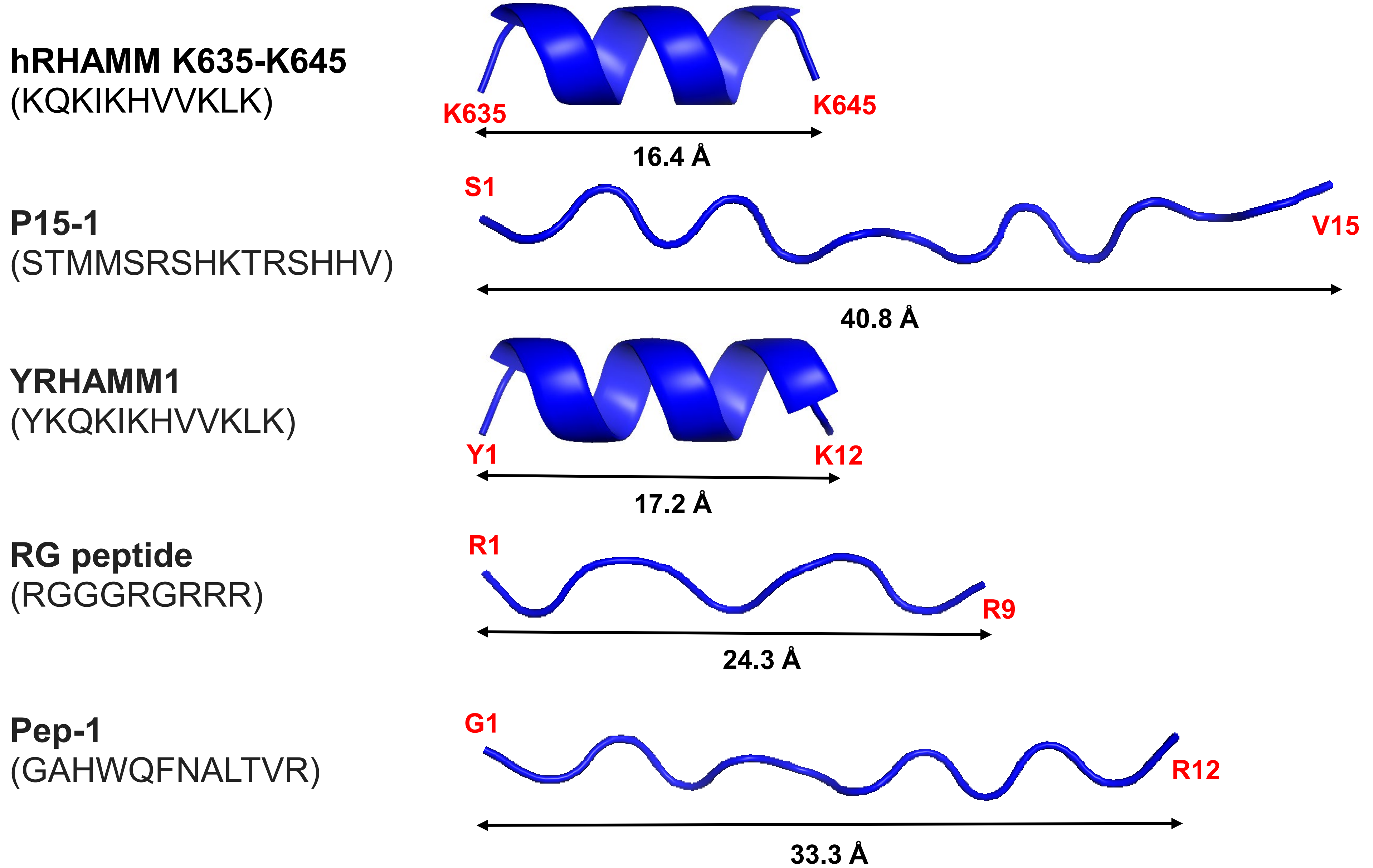

Figure S8. Comparison of the predicted backbone conformations for the human RHAMM K635-K645 peptide with other HA-binding peptides: P15-1, YRHAMM1, RG peptide, and Pep-1, all modeled using AlphaFold2 (Jumper, Evans et al. 2021, Varadi, Anyango et al. 2022). Structures depicted are the top-ranked models, with pLDDT scores of 84.4, 78.2, 84.1, 78.7, and 83.7 (top to bottom, respectively), corresponding to high (70-90) confidence level. All structures were visualized with PyMOL 3.0 software.

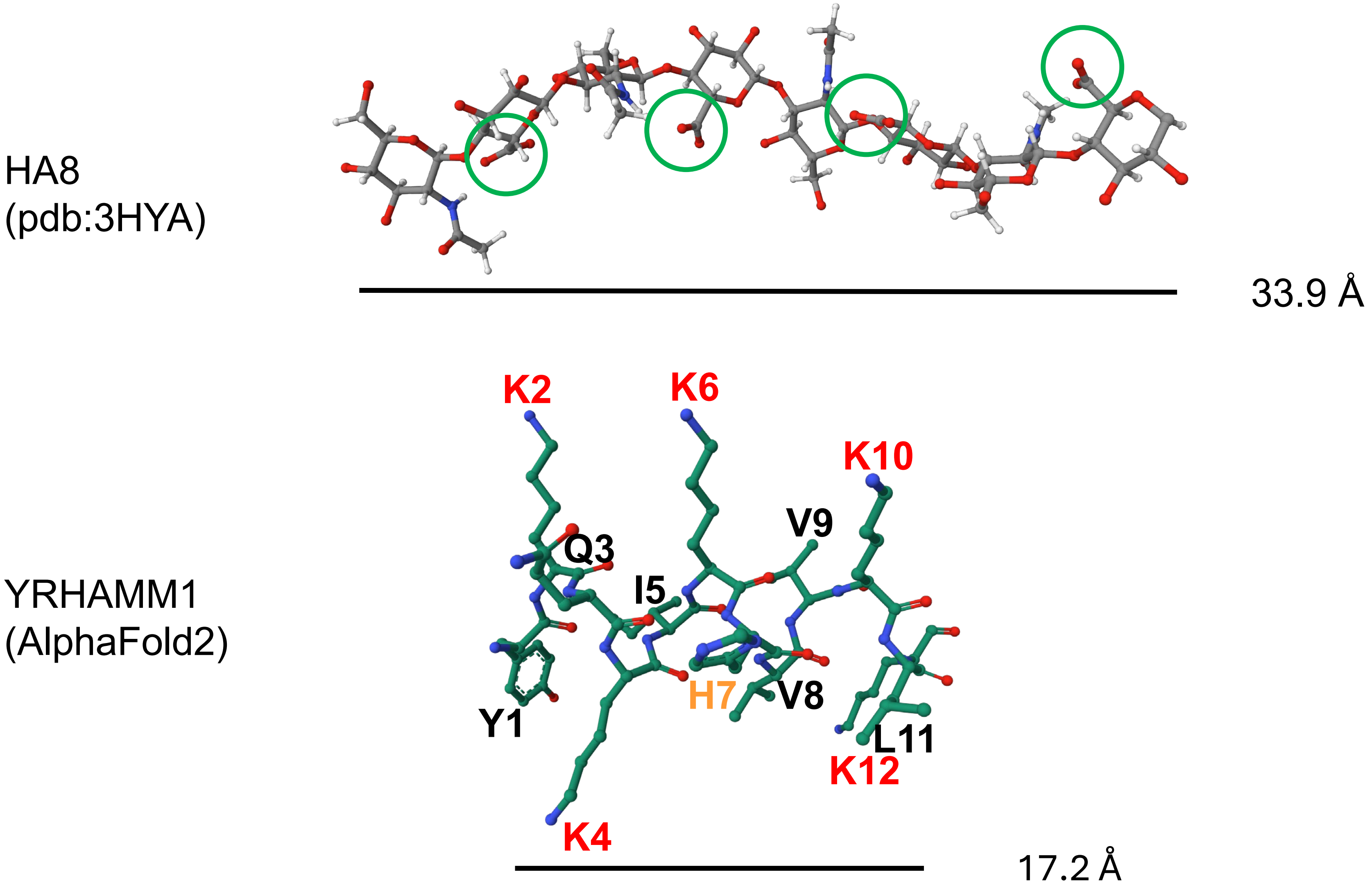

Figure S9. The top-ranked model for the YRHAMM1 peptide (same as hRHAMM635-645 with an added N terminal Y) adopts an alpha helical conformation, in which the spacing of basic amino acid side chains matches three consecutive carboxylates in HA. The peptide model is calculated using AlphaFold2. The basic K residues are labeled in red (orange for H). The HA octasaccharide (HA8) structure, determined by X-ray fiber diffraction analysis was downloaded from the RCSB PDB (rcsb.org), PDB ID 3HYA (http://doi.org/10.2210/pdb3HYA/pdb). All structures were visualized with Mol* software. The four carboxylate groups in HA8 are shown enclosed by green circles.

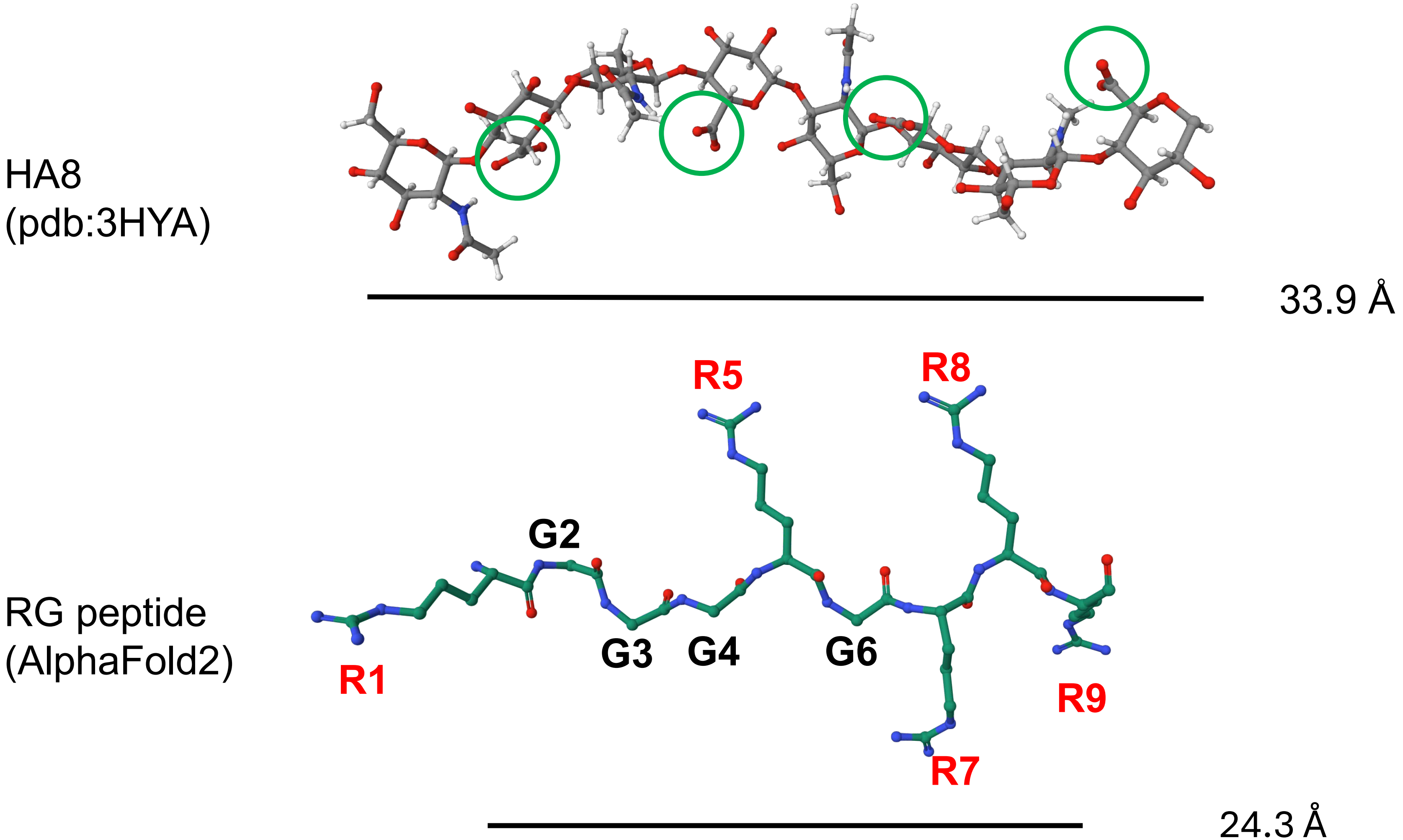

Figure S10. The top-ranked model for the rationally designed RG peptide (RGGGRGRRR) shows an irregular extended conformation. The basic amino acid side chains of the closely spaced R residues allow favorable electrostatic interaction with the carboxylates of HA. The peptide model is calculated using AlphaFold2. The basic R residues are labeled in red. The HA octasaccharide (HA8) structure, determined by X-ray fiber diffraction analysis was downloaded from the RCSB PDB (rcsb.org), PDB ID 3HYA (http://doi.org/10.2210/pdb3HYA/pdb). All structures were visualized with Mol* software. The four carboxylate groups in HA8 are shown enclosed by green circles.

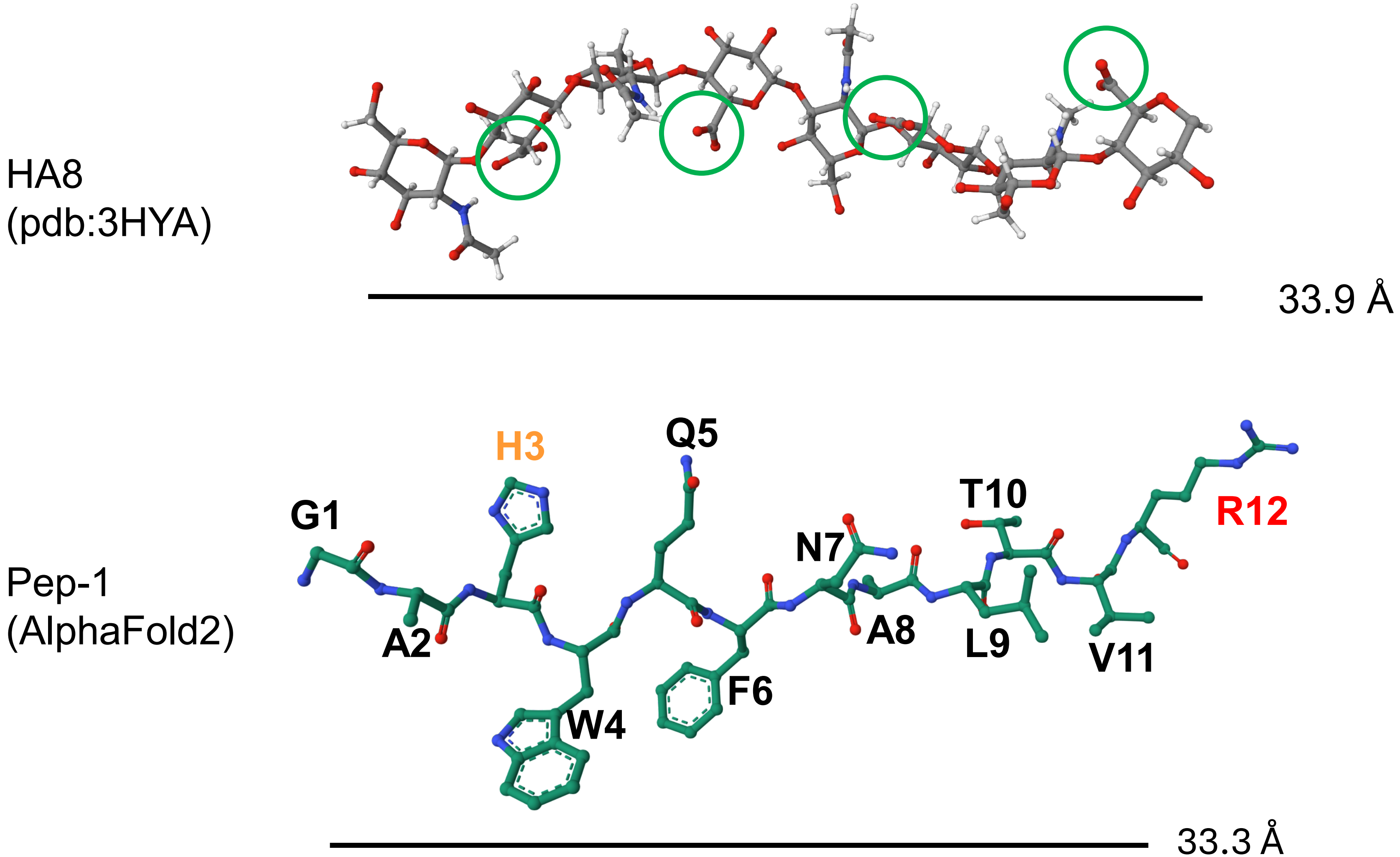

Figure S11. The top-ranked model for the Pep-1 peptide (GAHWQFNALTVR) shows an irregular extended conformation. There are only two basic amino acids, widely spaced, and the peptide binding to HA is not primarily electrostatic. The peptide model is calculated using AlphaFold2. The basic R residue is labeled in red (orange for H). The HA octasaccharide (HA8) structure, determined by X-ray fiber diffraction analysis was downloaded from the RCSB PDB (rcsb.org), PDB ID 3HYA (http://doi.org/10.2210/pdb3HYA/pdb). All structures were visualized with Mol* software. The four carboxylate groups in HA8 are shown enclosed by green circles.
